# Caries Management—The Role of Surface Interactions in De- and Remineralization-Processes

**DOI:** 10.3390/jcm11237044

**Published:** 2022-11-28

**Authors:** Jasmin Flemming, Christian Hannig, Matthias Hannig

**Affiliations:** 1 Clinic of Operative Dentistry, Medical Faculty Carl Gustav Carus, Technische Universität Dresden, Fetscherstraße 74, D-01307 Dresden, Germany; 2 Clinic of Operative Dentistry, Periodontology and Preventive Dentistry, Saarland University, D-66424 Homburg, Germany

**Keywords:** organic–inorganic interactions, acquired enamel pellicle, dental erosion, proteins, biofilm management, subsurface pellicle, demineralization, remineralization

## Abstract

Background: Bioadhesion and surface interactions on enamel are of essential relevance for initiation, progression and prevention of caries and erosions. Salivary proteins on and within initial carious and erosive lesions can facilitate or aggravate de- and remineralization. This applies for the pellicle layer, the subsurface pellicle and for proteins within initial carious lesions. Little is known about these proteinaceous structures related to initial caries and erosion. Accordingly, there is a considerable demand for an understanding of the underlying processes occurring at the interface between the tooth surface and the oral cavity in order to develop novel agents that limit and modulate caries and erosion. Objectives and findings: The present paper depicts the current knowledge of the processes occurring at the interface of the tooth surface and the oral fluids. Proteinaceous layers on dental hard tissues can prevent or aggravate demineralization processes, whereas proteins within initial erosive or carious lesions might hinder remineralization considerably and restrict the entry of ions into lesions. Conclusions: Despite the fact that organic–inorganic surface interactions are of essential relevance for de- and remineralization processes at the tooth surface, there is limited knowledge on these clinically relevant phenomena. Accordingly, intensive research is necessary to develop new approaches in preventive dentistry.

## 1. Introduction: The Role of Interfacial Phenomena in Cariology

**Figure 1:**
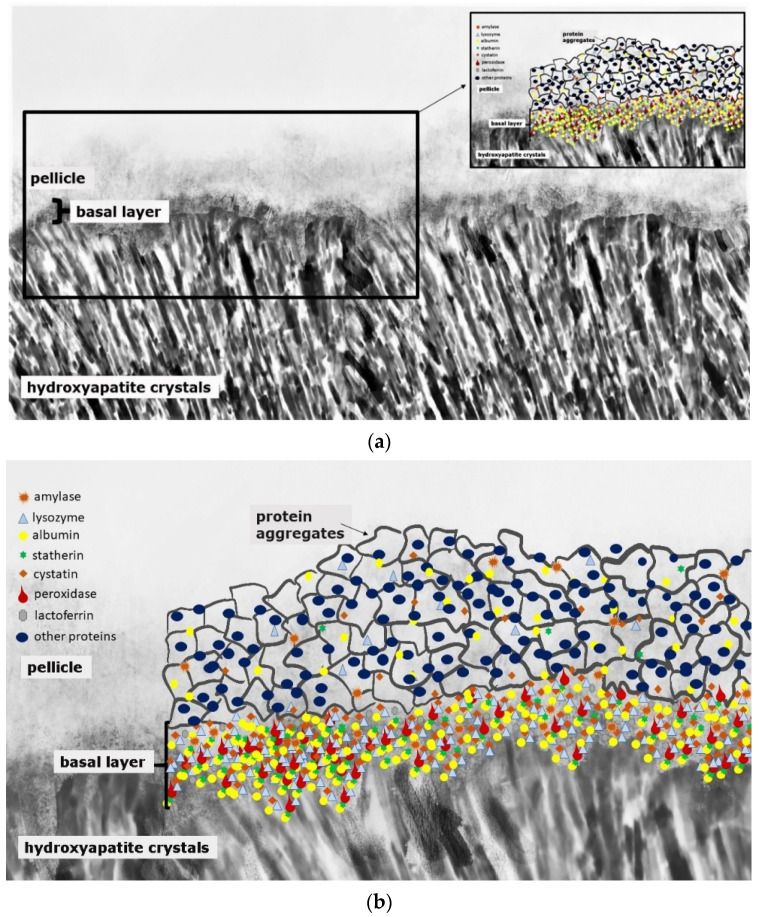
Schematic illustration of the acquired enamel pellicle (**a**,**b**). The primary basal pellicle layer consists of pellicle precursor proteins such as statherin, histatin, acidic proline-rich proteins (PRP), mucins, amylase, cystatin, peroxidase, lysozyme and lactoferrin (**b**). After the formation of this dense protein network of the basal layer, the pellicle maturation process is characterized by the adhesion of 100–200 nm sized protein aggregates and peptide complexes (**b**).

**Figure 2:**
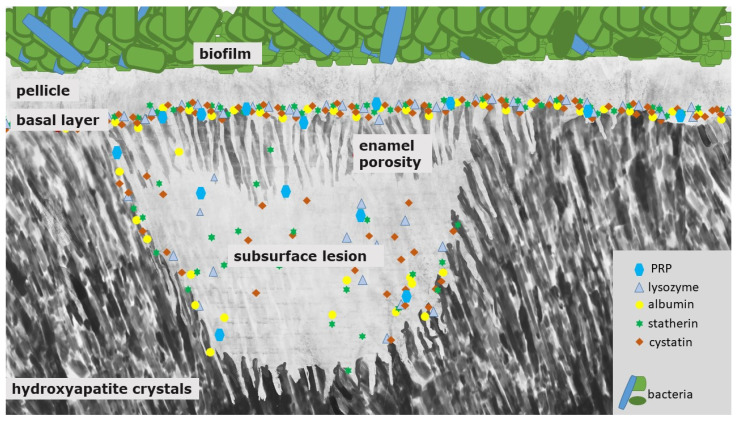
Schematic illustration of a white spot lesion. It is characterized by a subsurface demineralization with an increased enamel porosity and porous outer tissues beneath. First, PRP and other components with inhibiting abilities protect the surface from further demineralization and also prevent crystal growth. Due to their size, they can only enter and diffuse into the deeper enamel layers at an intermediate level of caries lesion progression. At that state, the pores are wider, and small pellicle precursor and acid-resistant proteins might also be found inside the subsurface lesion. Consequently, differently sized proteins remain at different levels of the lesion, which they coat quickly during the demineralization process.

**Figure 3:**
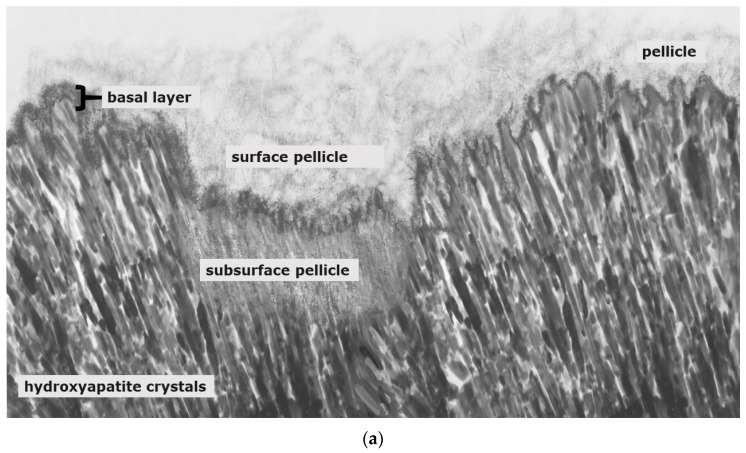

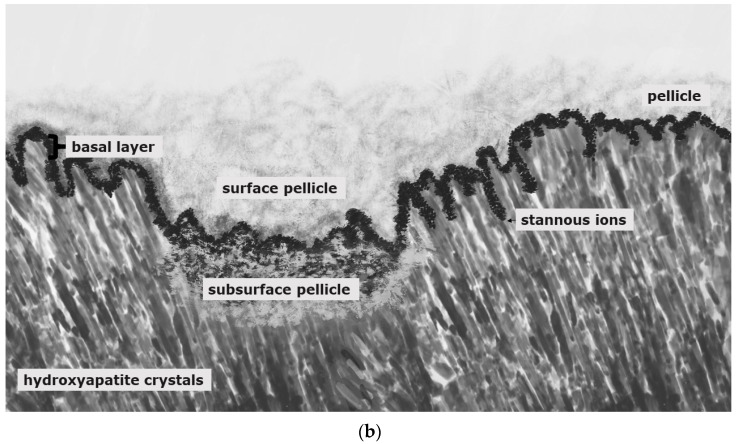
Schematic illustration of pellicle samples 8 h after a mild erosive attack ((**a**); e.g., agents with pH-values below 5) and after application of stannous-containing agents with low pH-values ((**b**); pH-values below 5). Mild erosive attacks lead to the formation of areas with a subsurface pellicle (**a**,**b**). Thereby, demineralized areas are filled with proteinaceous structures. A basal, more compact protein layer, the so-called basal layer, can be found in direct contact to the hydroxyapatite crystals (**a**,**b**). In contrast to other fluoride containing mouth rinses (**a**), agents with stannous ions show accumulations of stannous ions at the hydroxyapatite surface and inside the subsurface pellicle (**b**).

Caries and erosive mineral loss are still highly prevalent pathological processes in dentistry [1]. Modern concepts in conservative dentistry aim at remineralization and consolidation of such lesions; invasive therapy with fillings should be avoided or at least delayed [2]. Prerequisites for this approach are highly sensitive diagnostic techniques for the detection and monitoring of initial lesions as well as effective preventive agents and a profound understanding of the processes occurring at the surface and in subsurface layers at a nanoscopic level [2]. At first glance, it seems as if these topics have been explored completely and are the content of textbooks. A closer look at different aspects indicates that several key features are still not fully understood. This applies especially for the interactions of organic and inorganic structures directly underneath the surface. The following literature search was conducted using the PubMed and Medline databases as well as Google Scholar. Due to the unique conditions in the oral cavity, in vivo or in situ studies were mainly included in the review. In vitro data were considered if the experiments provided essential information on mineralization processes. This applied especially for methodical approaches not applicable in situ or in vivo, respectively. Bioadhesion and surface interactions on dental enamel are of essential relevance for initiation, progression, consolidation and prevention of caries and dental erosions. Salivary proteins on and within initial carious and erosive lesions can facilitate or aggravate de- and remineralization [3,4]. This applies for the pellicle layer (Figure 1), for proteins within initial carious lesions (Figure 2) and for the subsurface pellicle in superficially demineralized enamel (Figure 3). Despite this fact, little is known about the composition, function and ultrastructure of these proteinaceous structures related to initial caries and erosion.

In this context, there are relevant terms illustrating the processes occurring at the interface of the enamel and the oral fluids: The proteinaceous pellicle layer is formed on dental hard tissues. The following initial carious demineralization is characterized by a pseudo-intact surface [5]. Finally, pores on and in the enamel are penetrated by proteins having a very high affinity to etched enamel with a high surface energy. In contrast, erosive attacks lead to a complete demineralization of the enamel’s surface structure with limited possibilities for remineralization [6].

## 2. From Initial Biofilm to Initial Carious Lesion

Caries is an infectious disease of bacterial origin. It is the outcome of a caries pathogenic biofilm, the so-called dental plaque [7,8,9]. To start biofilm formation on solid surfaces, microbiota have to adhere to the acquired enamel pellicle layer [10,11].

This organic surface layer starts to form instantaneously after the contact of salivary proteins, glycoproteins and lipids with the non-shedding tooth surface or dental materials, respectively [12]. Thereby, the proteomic composition of the early (3-min) pellicle layer shows an individual pattern and is characterized by the favored adsorption of low-molecular weight proteins [3,13,14]. This includes a highly selective and dynamic adsorption process of salivary proteins and peptides, macromolecules from gingival crevicular fluid, blood, bacteria, mucosa and diet [11,12,13,15,16]. Figure 1 represents a schematic illustration of the acquired enamel pellicle. The primary basal pellicle layer consists of pellicle precursor proteins such as statherin, histatin and acidic proline rich proteins (PRP) (Figure 1) [17]. They attach via their calcium-binding domains, within seconds to minutes, to the tooth’s surface and represent the initial state of the pellicle formation process [13]. Moreover, these proteins maintain the mineral homeostasis by having a high affinity to calcium ions and, therefore, to hydroxyapatite [18,19]. Consequently, early pellicle formation is characterized by the interaction of calcium-binding proteins with the inorganic components of the enamel layer. After the formation of this dense protein network of the basal layer with further proteins such as amylase, cystatin, lysozyme and lactoferrin, the further pellicle formation process is characterized by the adhesion of 100–200 nm sized protein aggregates and peptide complexes (Figure 1). In addition, high-molecular weight proteins such as mucins adhere later to the pellicle layer in the maturation state [17,20].

Thereby, the proteomic profile of different individuals shows a high variability [3]. Nevertheless, a basic core set of 68 proteins was identified and represents proteins with antibacterial (lysozyme C, lactotransferrin, peroxidase, cystatin), lubricating (mucin 7), protein–substrate- (protein S-100 family, annexin A1, elongation factor 2), protein–protein- (14-3-3 protein family, protein–glutamine–gamma–glutamyltransferase E) and pellicle-integrity-promoting functions after 3 min of pellicle formation [3,14]. This core set of proteins was analyzed in the studies of Trautmann et al. and Delius et al. on ceramic specimens and in the study of Trautmann et al. on bovine enamel [3,13,14]. An accordance of 91% between the study of Delius et al. and the study of Trautmann et al., as well as 100% between the two studies of Trautmann et al., was observed [3,13,14]. Consequently, these proteins adhere universally and irrespective of the underlying substrate. Therefore, they can be considered as omnipresent proteins and a core set of the 3-min pellicle layer.

Analyses of the 5-min, 10-min, 60-min and 120-min pellicle proteome revealed a change in quantity and quality of the pellicle proteins during pellicle maturation [21]. Thereby, cystatin S and SA were increased between 10 and 60-min of pellicle formation and reduced after 120 min of pellicle formation. Additionally, acidic proline-rich proteins (aPRP) were relatively increased (rate: 137%) after 120 min [21]. These proteins are the key players in mineral homeostasis at the tooth surface [22].

Further salivary proteins such as α-amylase, mucin 5b, lysozyme and peroxidase showed a significant increase during pellicle development and maturation in vivo [21]. They interact with other proteins. Consequently, that explains the high increase in these proteins during the process of pellicle formation and, especially, in the later stages of pellicle maturation [21,23,24]. 

Proteomic analyses of the 120-min pellicle identified 15 core proteins. Among them are different isoforms of α-amylase (1A, 1B, 1C), annexin A1, BPI fold-containing family A member 2, carbonic anhydrase 6, cathepsin G, cystatin S, glyceraldehyde-3-phosphate dehydrogenase, immunoglobulin heavy constant alpha 1 and kappa constant, lactotransferrin, prolactin inducible protein and protein S100–A8 and A9 [25]. Among these 15 proteins, 11 proteins were enriched in biological processes such as antibacterial and antimicrobial humoral response pathways and regulation of apoptotic and other defense processes [25].

Consequently, the pellicle contains several defense mechanisms and relatively high amounts of lysozyme and peroxidase [11]. Nevertheless, ligands and bacterial receptor sites can be found in the pellicle layers (glycolipids, fibrinogen, collagen) that favor the initial bioadhesion of species such as streptococci and actinomyces [11,26,27]. After the early stages of bioadhesion, the interbacterial connections lead to the formation of the dental plaque. The detailed composition of oral microbiota is determined to be multifactorial. It was shown that it depends on the intraoral localization of the oral biofilm and also shows a high variability among different individuals [28]. The composition of the initial biofilm after 2 h in situ already showed a highly complex composition [28]. After 6 h, 90 bacterial species from 40 genera and 7 phyla were identified in vivo [11,29]. Relevant microorganisms in caries lesions of adults are lactobacilli, actinomyces, bifidobacteria, veillonella, cutibacteria and atopobium sp., as well as propionibacterium acidifaciens [30,31,32,33]. Thereby, dietary habits significantly determine the composition of the oral biofilm [34]. High sugar intakes shift the ecological equilibrium to an enhanced colonization with oral pathogens. This pathogenic condition is reflected by the high amount of organic acids produced by the bacterial digestion of carbohydrates, ultimately resulting in caries [8,35,36,37,38]. Extracellular polymeric substances (EPS) have a strong role in the development of the pathogenic biofilm [39], regulating the tolerance towards antibacterial agents and mediating bacterial adherence to the tooth surface [8,9,39,40,41,42]. A biopolymer matrix is formed during this process and improves the mechanical stability of the bacterial biofilm, regulating diffusion of chemical substances into the biofilm [43,44]. The shift from a physiological equilibrium to a pathogenic state is influenced by local acid production [45,46].

## 3. Proteins in Initial Carious and in Initial Erosive Lesions—The Subsurface Pellicle

Mature healthy enamel contains very low amounts of proteins (less than 1% by weight): amelogenin, ameloblastin, enamelin, albumin, hemoglobin subunits, collagens, anthithrombin-III and alpha-1-antithrypsin have been identified by several research groups [47,48].

Proteinaceous layers on dental hard tissues can prevent or aggravate demineralization processes, whereas proteins within initial erosive or carious lesions might hinder remineralization considerably and restrict the entry of ions in carious lesions [49,50]. In vitro experiments clearly indicated that deproteinization facilitated mineral uptake [49]. This applied for carious as well as erosive lesions in model systems. However, there is very limited knowledge on proteins within initially demineralized enamel in vivo. Older ex vivo studies indicated that exogenous proteins are present in demineralized enamel of white spot lesions. Amylase and albumin were detected by gold-immuno-labelling [51]; the amino-acid composition of proteins in experimental white spot lesions was characterized in a well-designed in situ study, and ex vivo samples from extracted teeth served as a reference [52]. To the best knowledge of the applicants, data gained with modern proteomic methods are lacking despite the fact that methods for proteomic analyses of tooth enamel are well established [48] and can be combined with clinically relevant in situ models [52,53,54].

## 4. Differences between Erosion and Carious Lesions in De- and Remineralization

An initial carious lesion results from a predominance of demineralization processes driven by bacterial biofilms and their acid production. Consequently, a white spot lesion can be detected (Figure 2). Clinically, it is indicated by a white enamel surface. In the following, colorants from natural substances such as teas, polyphenols or coffee or from substances such as tobacco can become deposited into this demineralized surface—the so-called brown spot lesion [5].

The white spot lesion is characterized by a subsurface demineralization with an increased enamel porosity and porous outer tissues beneath (Figure 2) [5]. The classical histological zones of the white spot lesion are, thereby, the translucent zone (in contact to the unmodified, healthy enamel; pores 1%), the dark zone (pores 2–4%) and the body of the lesion (zone with the highest mineral loss: 25%, pores 5–25%) [55]. The mineral dissolution can be explained by an under-saturation and formation of fluorapatite in the surface area of the enamel layer [5,56]. Salivary proteins such as PRP and other components with inhibiting abilities protect the surface from further demineralization and also prevent crystal growth (Figure 2) [5]. It was shown that these proteins can also inhibit the remineralization process of initial carious lesions [51].

In order to prevent an exuberant precipitation of calcium-phosphate, proteins from the family of proline rich proteins (PRP) can be found at the tooth surface. They prevent precipitation of calcium-phosphates and have a good adsorption at the tooth surface. Although statherins and PRPs inhibit these precipitation processes in saliva and at the tooth surface, initial caries lesions can remineralize to a certain extent; this is due to the size of these inhibiting proteins. In the initial states of the caries lesion, these proteins cannot diffuse through the relatively small porosities of the semi-intact surface layer of an initial carious lesion, while the small calcium and phosphate ions as well as stannous ions from fluoridation agents can easily diffuse through these porosities at the surface of an initial lesion. Thereby, the scale of the proteins ranges between 1 nm to 4 nm (i.e., statherin: ~2 nm, cystatin: ~3 nm) and the size of the calcium, phosphate and stannous ions ranges between 69 and 95 pm (ionic radii of stannous ions) [57,58]. Consequently, the precipitation-inhibiting effect of these proteins is not decisive in initial carious lesions as long as the pores are small enough. 

Therefore, PRPs might not diffuse into the deeper enamel layers and, thereby, adsorbed proteins seal the pores in the initial states of the caries’ developing process (Figure 2). Consequently, their protective abilities can only stabilize the outer surface area [5].

Clearly, proteins may have a crucial role in the progression of caries lesions due to an accumulation of organic material within the lesion over time [52,59]. Proteins might close the porous structure of the lesion on the tooth surface and, thereby, restrict the diffusion of further proteins, fluorides or other molecules into the lesion (Figure 2) [60]. At the later states of caries lesion progression, they can probably promote remineralization to a certain extent, but we assume that the diffusion of proteins into the lesion is not reversible, therefore a restitutio ad integrum cannot be achieved. 

Serum albumin seems to adhere to the tooth surface and has inhibiting effects on crystal growth [61,62]. Since Robinson et al. have shown by SEM–immunohistochemistry that albumin can distribute within white spot and fissure lesions and was rather not present in sound enamel [60], Shore et al. investigated the distribution of serum albumin and salivary amylase within white spot lesions related to different states of demineralization [51]. Interestingly, serum albumin and salivary amylase were detected in areas with 10–20% demineralization [51]. Pathohistologically, that represents a region between the dark zone and the body of lesion [51]. The authors assume that the accumulation of these proteins in the region of the lesion is due to multiple factors such as “pore size, crystal surface area available for binding and also pH (i.e., proton concentration)” [51]. These proteins show a high affinity to hydroxyapatite at low pH-levels. Although zones with 20% demineralization are nearer to the low pH-levels, these zones are also accessible for the salivary buffering capacity and protein retention might only be transitional [51]. On the contrary, areas with 10–20% demineralization show higher pH levels, but they also undergo less buffering from saliva, resulting in the main retention area for proteins such as albumin.

These studies show that the presence of proteins in specific areas and states of the caries lesion has a major impact on lesion progression. Therefore, it is surprising that since the release of the above-mentioned studies, there are no further or current studies examining the protein content of carious lesions with new approaches such as proteome analyses. We assume that inside the subsurface lesion, smaller pellicle precursor and acid-resistant proteins might be detectable (Figure 2). Due to their small size (<3 nm) they could diffuse through the pores into the lesion (Figure 2). Nevertheless, this assumption needs to be verified by further studies.

Furthermore, the size and direction of the initial lesion is influenced by the microbial colonization on the surface and the prism direction [5]. Below the proximal area of the tooth, the lesion is oval shaped. Inside the lesion, the mineral solution follows the direction of the prisms. The highest activity of dissolution can be found along the central traverse [5]. The histological image of the lesion mirrors the single stages of the progressing lesion, influenced by the dynamics of the biofilm on the enamel surface [5,63].

In contrast, erosions are characterized by mineral loss caused by acids that do not emerge from bacteria. These acids are of endogenous or exogenous origin [64,65]. Erosion results from prolonged acid influence if the surrounding area is under-saturated with minerals [56]. The pellicle layer has erosion-protective properties, but due to post-industrial eating habits, the physiological pellicle layer is no sufficient protection against these acid influences [66]. If there is a prolonged acid influence on the enamel surface, erosive lesions can be detected [67]. Initial carious lesions are characterized by the subsurface lesion, as described above. On the contrary, erosive lesions proceed centripetally and the area with the highest mineral loss is peripheral [68]. The erosive lesion in the dentin is characterized by a demineralization of the peritubular dentin and further by the intertubular areas [69]. The loss of inorganic dentin components results in a residual dentin matrix that prevents the further mineral loss and slows down the process of demineralization [70].

**Figure 4:**
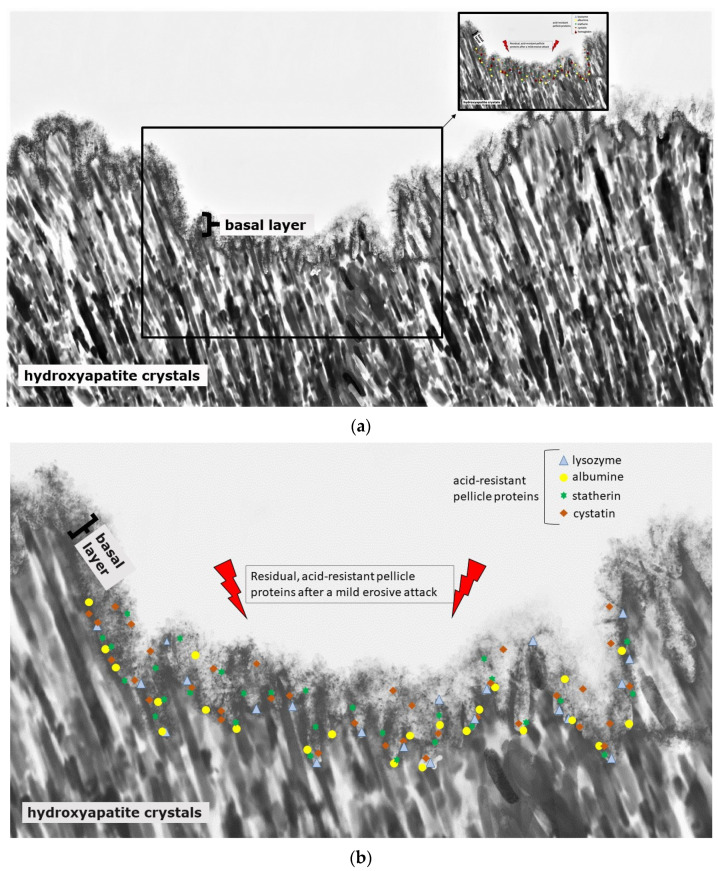
Schematic illustration of the protein accumulation and infiltration directly after a mild erosive attack (**a**,**b**). Acid-resistant proteins such as lysozyme, cystatin, statherin and albumin remain in the basal layer at the hydroxyapatite surface of the subsurface lesion (**b**). Other non-resistant proteins dissolve. The loosened proteinaceous network above the basal layer is removed by the acidic attack, and proteins can enter into deeper enamel structures (**a**,**b**).

Initial carious lesions can be demineralized because of their subsurface lesion and their porous enamel layer (Figure 2); erosive lesions, on the contrary, only demineralize in their initial state because of the degradation of the complete enamel surface (Figure 4).

## 5. Proteins in the Oral Cavity of Caries-Active and -Inactive Subjects 

### 5.1. Differences in Salivary Protein Composition in Caries-Active and -Inactive Individuals

Since most proteins that interact with re- and demineralization processes in carious lesions have their origin in saliva, it was assumed that caries-active and -inactive individuals would show distinct differences in their protein composition.

Protection mechanisms against caries and the support of maintaining tooth integrity are performed by salivary proteins through the following actions: Formation of the pellicle layer and its acid resistance (aPRP, statherin, cystatin S, carbonic anhydrase I, II, VI, histatins), prevention of demineralization and promotion of remineralization (aPRP, statherin, histatins) as well as antimicrobial properties (lactoferrin, lysozyme, peroxidase, α-amylase, sIgA, IgG, statherin, histatins, mucin 5CB, C7, cystatin) [17,71,72].

PRP, statherin and cystatin, especially, are the proteins that are responsible for mineral homeostasis and binding to calcium-phosphates of hydroxyapatite. Therefore, it is necessary to evaluate the specific role of these important key player proteins in the caries’ developmental process in further studies. Schweigel et al. examined the proteome of the acquired salivary pellicle and saliva in general. Regarding their physico-chemical properties, they did not differ much [16]. Interestingly, molecular size distribution especially varied clearly. Thereby, pellicle proteins were significantly smaller and shorter than salivary proteins [16]. This size distribution is a crucial factor in terms of process regulations such as caries initiation and progression. Especially with regard to the affinity of proteins to the enamel surface, a higher level of phosphorylation and glycosylation was observed in pellicle proteins [16]. Moreover, pellicle proteins showed a higher buffer capacity in very acidic environments [16]. Consequently, these pellicle proteins are able to protect the hydroxyapatite surface against acidic attacks by an increased ionic interaction strength, and, due to their small size, they can diffuse easily through the enlarging size of the porous enamel structure of developing carious lesions (Figure 2) [16]. Salivary proteins do not need these properties. The salivary buffering capacity is maintained through soluble salivary ions [16]. Salivary proteins need protection against denaturation and decreased affinity to enamel surfaces, which is maintained by larger scale salivary proteins [16].

### 5.2. Differences of Pellicle Protein Composition in Caries-Active and -Inactive Individuals

An impact of the pellicle proteome in caries-active and -inactive individuals was expected due to significant differences in the salivary proteome that serves as the origin of pellicle proteins [73]. Studies of the 3 min pellicle proteome showed that pellicle formation is a highly selective and quick process. As a result, pellicle proteins and enzymes accumulate at the tooth surface (Figure 1) [14]. The pellicle shows a physiological activity already after a few minutes [14,74]. 

Trautmann et al. identified n = 1188 different pellicle proteins on in situ worn ceramic specimen [3]. There was no significant difference in the absolute and relative abundance of pellicle proteins in caries-active and -inactive individuals. Accordingly, specific proteins could not serve as caries biomarkers. Nevertheless, n = 320 proteins were solely found in the caries-active group, and n = 116 proteins were only detected in the caries-inactive group; n = 752 proteins were analyzed in both groups [3]. Still, no protein was found that was detected in all individuals of the caries-active or -inactive group. Consequently, they cannot serve as caries biomarkers [3]. Results of a gel electrophoresis analysis performed by Vitorino et al. could not be confirmed by the results of the proteome approach of Trautmann et al. [3,75]. Vitorino et al. suggested that cystatin isoforms (S, SA, SN) can be detected solely in caries-inactive individuals [75]. Instead, Trautmann et al. detected these specific isoforms in the 3-min pellicle of caries-active and -inactive individuals [3].

### 5.3. Qualitative Pellicle Proteome Composition in Caries-Active and -Inactive Individuals

The protein functions of specific proteins can be summarized in different molecular functions (MF). The most abundant MF in caries-active individuals are associated with binding abilities, such as protein-binding, cell-surface-binding and ion-binding abilities [3]. These binding abilities indicate an enhanced bacterial interaction resulting in the promotion of biofilm formation. In contrast, the most abundant MF in caries-inactive individuals have enzymatic abilities, promoting a higher defense and caries prevention [3].

### 5.4. Quantitative Pellicle Proteome Composition in Caries-Active and -Inactive Individuals

The quantitative proteome analysis of Trautmann et al. analyzed 23 potential caries biomarkers via statistical analysis [3]. An unadjusted quantitative analysis was, thereby, performed based on the emPai of the identified proteins [3].

Another proteome analysis of caries-active and -inactive individuals evaluated the pellicle proteome after 5- and 120-min pellicle formation [76]. They identified n = 264 proteins [76]. Within their study, they detected differences in mucin 7, lysozyme C and immunoglobulins between the active and inactive group [76]. Mucin 7 and 5b belong to caries-protective proteins of the oral cavity [77]. A correlation of these proteins with the caries status is, so far, not verified [78,79,80]. Additionally, Luo et al. showed an increase in IgA, annexin A1 and neutrophil defensin 3 in the 5- and 120-min pellicle of caries-inactive individuals [76]. In this study, pooled pellicle samples were used to perform the analyses. In contrast, Trautmann et al. and Delius et al. performed individual pellicle proteome analysis, and the above-mentioned proteins could not be confirmed as individual biomarkers. They detected these specific proteins in the basic protein profile of their examined individuals after 3 min of pellicle formation [3,14]. Consequently, further studies with larger cohorts without pooled samples need to be performed.

## 6. Gold Standard in Preventive Dentistry—Fluorides—Models for Preventive Effects

Since the 1950s, fluorides have been cornerstones of individual and group prophylaxis against caries [81,82,83,84,85]. Thereby, fluorides in their topical application form are still the gold standard in the prevention of caries and demineralization such as erosive tooth wear [86,87,88]. Topical fluoridation with highly concentrated fluoride content and low pH might protect against these diseases by forming a calcium-fluoride or calcium-fluoride-like layer on the tooth surface [89,90,91,92,93,94,95]. Current models related to the fluorides’ mode of action insufficiently describe the precise details of how fluorides could work. However, there is strong evidence on the basis of clinical studies that fluorides are effective in preventing caries and erosive tooth wear [82,96].

Fluoride reservoirs can be divided into the mineral calcium fluoride, such as precipitates, and biologically/bacterially bound calcium fluoride deposits [97].

It is assumed that the pellicle influences the formation and stability of calcium-fluoride-like layers [98]. Thus far, the interaction of pellicle proteins and calcium fluoride, such as precipitates, has not been investigated, although it is assumed that pellicle formation occurs above these precipitates. The amount of these layers and precipitates is dependent on the pH-value, the concentration, the time and the frequency of the applied fluorides [94].

Calcium-fluoride-like precipitates can act as a barrier against acidic diffusion into the dental hard tissues below [99,100]. At neutral pH-values, these precipitates are expected to be protected from quick dissolution by the adsorption of secondary phosphate to calcium binding sites in the surface of calcium fluoride crystals [101]. During attacks with acids from cariogenic microorganisms, the primary phosphate is predominant. Consequently, the inhibition of dissolving calcium is not possible and is followed by the release of fluoride ions due to the reduced concentrations of secondary phosphate ions [101]. Therefore, calcium-fluoride-like precipitates are expected to represent an efficient source of free fluoride ions during acidic attacks [94,101]. While, in theory and in vitro examinations, these processes are plausible, the in vivo implications of these calcium-fluoride-like precipitates are still not clear [101]. Additionally, it would be interesting to see which role proteins of the mineral homeostasis such as aPRP, histatins, cystatin S play in the formation and maintenance of calcium-fluoride-like precipitates. This fundamental question should be the subject of further studies to gain an understanding of organic and inorganic interactions at the tooth surface.

Additionally, studies showed a major impact of fluorides on the biological activity of *S. mutans* in its planctonic state and in its biofilm community (less gene expression of metabolic pathways) [102,103,104]. Thereby, an inhibition of enolase and other enzymes of the glycolytic pathway was observed [105]. Consequently, the bacterial cytoplasm will be more acidic [106]. Additionally, fluorides can lower the cariogenicity of the biofilm community by reducing the acid production and the EPS–matrix-synthesis [102,107]. Over decades, it was assumed that the biofilm is a possible fluoride reservoir [108,109]. These effects and intraoral surface interactions have been examined mainly in vitro [106]. A current in situ study could not confirm that fluoride-rich biofilms can release fluorides if the pH-value is low [110]. 

Toothpastes contain fluorides either as monosubstances or in combinations of the inorganic sodium fluoride, sodium monofluorophosphate, stannous fluoride or the organic amine fluoride, respectively [111,112]. 

The majority of commercially available rinsing solutions contain inorganic sodium fluoride or organic amine fluoride [96,113,114,115]. Previous studies could not yield clear evidence of whether sodium or amine fluoride are more protective on the tooth surface [113,116,117]. Two in situ studies with fluoride monosubstances recently examined that inorganic stannous fluoride and stannous chloride-containing solutions appear to be the most effective types of monosubstances to prevent erosive enamel demineralization and bacterial adhesion on the tooth surface [118,119]. The observed effects primarily have to be attributed to the stannous ions [120,121,122]. 

Stannous fluoride, thereby, has two characteristics: On the one side, it facilitates the fluoride adsorption and thus improves enamel remineralization and stops demineralization [123]. On the other side, stannous ions are able to inhibit plaque acidogenicity by interrupting membrane transport systems and enzymes that are involved in glycosidic pathways [124]. The uptake of stannous ions into bacterial cells changes bacterial growth and the bacterial metabolism leading to cell death after integration of the ions [124,125,126]. For a long time, the impact of stannous fluoride on initial bacterial colonization referred to two older studies [127,128]. Current TEM-investigations have shown that stannous ions appear to enhance the pellicle’s resistance against bacterial colonization and acidic dissolution in 8-h and 30-min in situ pellicle samples after rinsing with stannous fluoride and stannous chloride [118,119]. Thereby, the basic layer of the 30-min and 8-h in situ pellicle revealed, in part, a higher electron density with a verifiable accumulation of stannous-compounds that also persisted after incubation in HCl (Figure 3b) [118,119]. 

The prevention of erosive tooth wear is due to a deposited, stable and acid-resistant stannous layer on the tooth surface. Caused by the low pH of the solutions, an incorporation of stannous ions into the enamel tooth structure forms this stannous layer (Figure 3b). Erosive attacks with mild agents with pH values between 3.5 and 5 lead to an accumulation of stannous ions at the hydroxyapatite surface and a protein accumulation above this stannous-containing layer (Figure 3b). Consequently, inorganic stannous ions lead to a modification of the subsurface pellicle (Figure 3a,b).

It can be assumed that stannous ions compete with calcium ions in similar binding points [123]. Stannous ions are not only important preventive substances, they also offer indications of how organic and inorganic interactions occur at the tooth surface. In preparation of TEM-images, stannous ions are not removed during the decalcification of the specimens. Therefore, stannous ions might serve as an indicator for ions during remineralization, such as calcium ions, since they become visible in TEM. 

In comparison to other fluoride cations, stannous-containing oral hygiene products seem to protect significantly better against erosive tooth wear [118,129,130]. 

Currently, there are two proteome studies evaluating the effect of different concentrations of inorganic sodium fluoride on proteinaceous structures (1, 2 or 5%) for two hours in vitro [131] and the effect of inorganic stannous fluoride on erosive tooth wear after 2 h (Sn (800 ppm/6.7 mM, SnCl_2_), F (225 ppm/13 mM, NaF), Sn and F combination (Sn & F)) [123].

The study of Siqueira et al. evaluated a total of 45 proteins in all groups; 12 proteins were exclusively present in the control group (deionized water), and 19 proteins were only present in the discs when treated with 5% sodium fluoride [131]. Proteins such as statherin and histatin 1 decreased with increasing levels of fluoride [131]. The study of Algarni et al. identified 72 proteins in all groups [123]. The pellicle that was treated with Sn & F showed a higher abundance for most of the identified proteins than the other groups. The examination indicated a reduction in enamel surface loss for Sn & F (89%) Sn (67%) and F (42%) compared to the control group with deionized water [123].

Further studies are necessary to understand the principles of the fluorides’ interactions with bacterial cells, salivary and pellicle proteins and the tooth surface. Thereby, research questions on the efficacy of fluoride-bound cation or the fluoride ion itself and how they interact with proteins need to be answered to gain a principle understanding of these oral health care products, that are highly efficient in daily oral health care [81,82,83,84,85]. Therefore, combinations of new microbiological approaches such as microbiome studies or proteome and metabolome studies need to be performed to gain knowledge of the complex mode of action. 

## 7. Prevention of Demineralization

One of the main physiological factors against demineralization of the tooth surface is the supersaturated oral fluid—saliva. Saliva and its buffer systems neutralize acids from the oral cavity and the tooth surface; besides, it contributes to the formation of the pellicle by providing essential pellicle proteins [132].

The acquired pellicle is formed after contact of the tooth surfaces with saliva and protects the tooth surfaces against acidic challenges. Thereby, the pellicle’s basal layer shows physiological protective properties towards acids by preventing a direct contact between acids and the tooth surfaces [133]. The pellicles’ protein network acts as a mechanical barrier against acidic influences and prevents the tooth from dissolution of calcium and phosphate ions [134,135,136]. Some pellicle components, such as cystatin B, are increased up to 20-fold after acidic challenges (citric acid; pH 2.5) [137]. Thereby, the pellicle acts as a modulating factor, aggravating and reducing the calcium and phosphate dissolution [138]. The thicker the pellicle layer, the more protection is detectable against demineralization [136,139]. The electron-dense basal layer of the pellicle yields the highest protection against acid attacks [134]. The exterior pellicle layers are of lower density and provide less protection [140]. 

Nevertheless, the protective function is generally limited due to the semi-permeable characteristics of the pellicle layer. Conclusively, frequent and severe acidic challenges lead to a partial or total loss of the pellicle layers and result in mineral loss of the tooth surface (Figure 3a and Figure 4) [134]. The pellicle proteome and peptidome can offer several indications as to which pellicle components contribute to the protective effects against erosive and bacterial acid challenges [141]. It was shown that specific components of the pellicle remain at the tooth surface, even after severe acidic events [133,140].

Some of these components, that are main parts of the pellicle’s basal layer, were identified by quantitative proteomic approaches [137,142,143]. Among the acid resistant proteins of the pellicle’s basal layer are cystatin [137], statherin, serum albumin [143] and hemoglobin (Figure 4c) [140,142]. In comparison with gastroesophageal reflux patients with erosive tooth wear, the hemoglobin-levels were elevated in the pellicle and saliva of gastroesophageal reflux patients without erosive tooth wear [142]. Conclusively, hemoglobin might serve as one of the protective components of the pellicle layer.

The adsorption processes change the secondary and tertiary structure of the pellicle enzymes and proteins [144]. As soon as they are immobilized in the pellicle layers, the active center underlies changes. Nevertheless, these enzymes and proteins are still in an active conformation inside the pellicle layers [145,146]. It is known that the pellicles’ precursor proteins are the most protective ones against erosive demineralization [74]. These proteins are, e.g., PRPs, cystatins, amylase, lactoferrin and histatins. Carvalho et al. assume that an increase in proteins with high affinity to hydroxyapatite in the pellicle’s basal layer could also lead to an increase in proteins binding to the pellicle’s precursor proteins [140]. 

**Figure 5:**
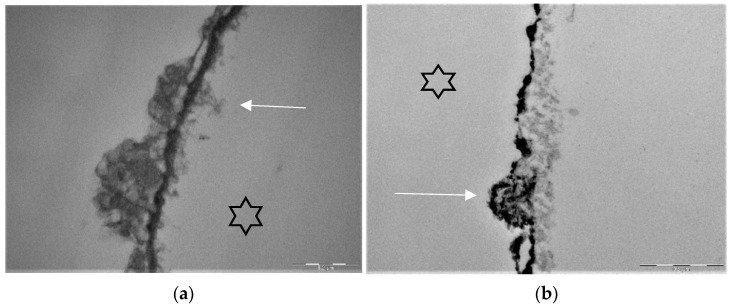
Representative TEM-images visualize the pellicle ultrastructure. A subsurface pellicle can be detected after rinsing with fragaria vesca extract (**a**) and stannous fluoride (**b**) after 8 h of oral exposition [119,147]. Demineralized areas are filled with proteinaceous structures (arrows) and stannous ions (**b**). A higher electron density of the pellicle’s basal layer can be observed after rinsing with stannous ion containing agents. The enamel was removed during specimen processing and the former enamel side is marked by an asterisk.

Subsurface pellicle: 

A special effect of demineralization on the tooth surface is a partially protein-infiltrated structure known as the subsurface pellicle. This phenomenon occurs after rinsing with acidic fluids, such as fluoride or polyphenol-containing rinsing solutions with pH-levels below 5 (Figure 5a,b) [119,147]. Thereby, the subsurface pellicle might represent a repair process.

Various models could describe this phenomenon. During the mild acidic attack, calcium ions and, later on, phosphate ions dissolve from their hydroxyapatite structure. The calcium ions are deposited at the tooth surface above, and the enamel surface becomes positively charged [74,148,149,150]. In the following, electrostatic interactions determine the adsorption processes between the residual acid-resistant proteins and the hydroxyapatite surface. Pellicle precursor proteins adhere via calcium bridging as well as non-covalent and hydrophobic interactions [74,148,149,150,151,152,153]. Thereby, cationicity and hydropathy are key factors that determine the selectivity and potency of proteins toward the anionic, negatively charged tooth surface. This involves statherin, histatin, acidic PRPs, mucins (5 and 7) amylase, cystatin, lysozyme and lactoferrin [74,144,145,154,155,156,157,158]. We assume, especially, that smaller and negatively charged proteins diffuse into the “porous, loosened” enamel structure and take place in the repair process. 

Thereby, statherin, histatin, cystatin and lactoferrin could be easily integrated into the loosened structure. Since they are also acid-resistant, they might as well interact with the remaining acid-resistant proteins via protein–protein interactions. Therefore, one remaining question is whether remineralization can occur through this remodeled protein network of the subsurface pellicle or if the subsurface pellicle represents a proteinaceous barrier against remineralization.

TEM images of our group show different findings after rinsing with stannous fluoride and an 8 h oral exposition time (Figure 5b). On the one hand, we find areas where no stannous ions are detectable at the bottom of the subsurface lesion (Figure 5b). On the other hand, there are areas where we can find stannous ions at the fundus of the subsurface lesions (Figure 3b and Figure 5b).

A proteome analysis could offer more information about this specific question and should be performed in future studies. It would be interesting to know whether stannous ions diffuse into the lesion after the acidic attack took place or if the stannous ions accumulate during the attack while rinsing the mouth with these mild acidic agents.

## 8. Impact of Proteins on De- and Remineralization

Proteins have a high impact on de- and remineralization processes on the dental hard surfaces. Particularly the proteins and peptides that form the pellicle layer play an important role for the non-shedding enamel surface. Due to the pellicle’s semipermeable characteristics, it prevents demineralization of dental hard tissues to a limited extend [66]. Further, the pellicle influences the adhesion of microorganisms and, later, on biofilm formation. This proteinaceous layer is, consequently, the mediator of all macro- and micromorphological interactions and has a crucial role in the development of diseases such as erosive tooth wear or caries. Therefore, its proteome and peptidome are of great interest [141]. On the basis of this developing knowledge, it is important to identify the role of proteins in interfacial processes and organic–inorganic interactions. It was shown that salivary proteins can adapt and respond to evolutionary pressure [159,160]. It was investigated that functional domains of the proteins were, thereby, included into their primary structure, resulting in higher activity [159,160]. Additionally, another evolutionary advancement is the formation of functional complexes built by salivary proteins in the oral cavity. These complexes serve as protection against proteolytic processes and lead to a distribution of these proteins on all surfaces of the oral cavity [160,161]. On this basis, current research tries to focus on identifying specific proteins and modifying their sequences, hoping that these new designed proteins are more efficient than evolutionarily developed physiological proteins of the oral cavity [140,141,160,162]. Currently, the investigations and development of such proteins are still at an early stage. Consequently, profound expertise for gaining knowledge in terms of applied basic research is needed to use bioinspired techniques for an optimized use of proteins. In conclusion, customized pellicle proteins and peptides are a novel preventive approach with versatile mechanisms of action, but so far, this is only based on the first in vitro results.

## 9. Promotion of Remineralization

At the moment, bioinspired pellicle engineering is being investigated by several study groups worldwide [140,141,160,162].

Thus, cystatin, histatin and statherin and their residues serve as bioinspired or modified molecules to improve the pellicles’ physiological properties against demineralization and promotion of remineralization in vitro [160,163,164,165]. These physiological proteins naturally exist in the oral cavity and are, therefore, biocompatible. The underlying idea is an advanced adsorption and enrichment of modified peptides into the pellicle layers and onto the enamel surfaces [160]. As a result, these proteins or modified peptides reduce the amount of enamel dissolution areas [160,166,167,168]. Additionally, these adsorbed proteins or peptides limit the acid transfer from biofilm communities onto the hydroxyapatite surface and vice versa, reducing the dissolution and diffusion of calcium and phosphate ions into the oral cavity [160,169].

Statherin modifies and regulates mineral formation. During pellicle formation, six peptides of the N-terminus regulate the binding to the hydroxyapatite surfaces [170,171]. These first six peptides were investigated by the in vitro study of Yang et al. [171]. They extracted and modified this peptide sequence and evaluated their enamel adsorption abilities and remineralization characteristics [171]. Under in vitro conditions, the peptide sequence (DpSpSEEKC) shows potential to adsorb to hydroxyapatite and remineralize the tooth surface [171]. The authors relate this effect to the peptide sequences’ carboxyl and phosphate groups and conclude that the peptide sequence DpSpSEEKC might be a suitable method to remineralize demineralized enamel surfaces [171]. 

Another in vitro study investigating pellicle engineering evaluated the effect of the nine--residue phosphopeptide derived from statherin, known as DR9 [163]. It was shown, that the enrichment of the pellicle layer with DR9 can lead to less mineral loss under in vitro conditions [163]. Overall, the main goal of pellicle engineering is the positive modification of the pellicle layer and an improvement in their protective properties against demineralization by erosive and bacterial derived acids. Therefore, histatin 3 and its functional 14-residue peptide (RR14) was studied by Marin et al. in vitro [160]. Additionally, they observed that the more phosphorylated the pellicle, the less dispersion of overlying biofilm was observed [160].

Overall, pellicle engineering shows promising results in vitro; further in situ and in vivo studies must take place to evaluate and assess the value of engineered proteins and peptides for future preventive strategies.

Besides pellicle engineering approaches, bioinspired remineralization becomes more and more relevant [172]. Several in situ studies indicate the use of polyphenols for protective pellicle modification [172,173,174]. 

## 10. Topics for Future Research

Future studies should focus on the role of proteins and peptides in the initial demineralization processes. Proteins could also serve as agents for the clinical application of initial caries lesions with specific “protein-cocktails”. Thereby, future studies should examine differences between acid-resistant proteins after erosive acidic attacks and bacterial acidic attacks. In that context, a proteome analysis of the subsurface pellicle would be necessary to detect differences. 

Additionally, the role of proteins and their influence on fluorides and vice versa had not been investigated so far. There is a very limited knowledge on organic and inorganic interactions at the tooth surface. In that context, the influence of different fluoride monosubstances on pellicle protein abundance would be very interesting. 

A further research question is whether fluoride cations can diffuse into the subsurface pellicle.

All things considered, the main central issue of these future research questions is, thereby, characterized and determined by organic and inorganic interactions in biomineralization during periods of de- and remineralization.

Future research questions:

- What is the diffusion time and characteristics of single proteins such as PrP, statherin and ions, like stannous ions, into carious lesions? How do pore sizes and lesion progression influence these diffusion processes?
- How can proteins close the pores of carious lesions? Which physicochemical processes might lead to such a sealing?
- Which protein content can be found in initial and severe carious lesions with proteome analyses?
- Which protein profile can be found in a subsurface lesion?
- What are the interactions between fluorides and proteins as well as fluoride-bound cations with proteins?
- How do engineered proteins and peptides affect preventive strategies in situ and in vivo?

## 11. Conclusions

Despite the fact that organic–inorganic surface interactions are of essential relevance for de- and remineralization processes at the tooth surface, little is known on this topic. Accordingly, intensive research is necessary to develop new approaches in preventive dentistry.

## Data Availability

No new data were created or analyzed in this study. Data sharing is not applicable to this article.
